# Asian Ethnic Group Differences in Formal and Informal Volunteerism Among Adults Aged 50 and Over in the United States

**DOI:** 10.1093/geroni/igaf122.1075

**Published:** 2025-12-31

**Authors:** Patrick Ho Lam Lai

**Affiliations:** The University of Oklahoma, Moore, Oklahoma, United States

## Abstract

As the aging population grows, volunteerism plays a vital role in promoting well-being and societal benefits. However, disparities in volunteerism persist across racial and ethnic groups, particularly among Asian Americans, a heterogeneous population often underrepresented or grouped homogenously in research. This study examines formal and informal volunteerism among Asian subgroups in the U.S. using data from the Civic Engagement and Volunteering Supplement of the Current Population Survey (2017, 2019, 2021). Focusing on adults aged 50-75, it investigates subgroup differences in volunteer rates and the interplay between socio-economic resources (income and education) and ethnic identity. Findings revealed significant differences across subgroups. Japanese, Koreans, and Filipinos were more likely to engage in formal volunteering compared to Chinese, while informal volunteering was higher among Filipinos and Asian Indians. Socio-economic factors, especially education, partly explained these disparities. Education had a less positive impact on formal volunteering for Koreans, Vietnamese, and other subgroups compared to Chinese, while income had a stronger influence for Filipinos. Informal volunteerism showed limited interaction effects with socio-economic resources, highlighting distinct dynamics between formal and informal engagement. This study emphasizes the importance of disaggregating data to better understand the unique experiences of Asian subgroups. Findings call for culturally responsive policies and programs to address barriers and promote equitable engagement opportunities for diverse midlife and older populations. Further research is needed to examine cultural and structural factors shaping volunteerism within and across Asian subgroups.

